# Recurrent Syncope, a Clue in Amyloid Cardiomyopathy

**DOI:** 10.1155/2018/1864962

**Published:** 2018-01-28

**Authors:** Julian A. Marin-Acevedo, Catalina Sanchez-Alvarez, Ali A. Alsaad, Ricardo J. Pagán

**Affiliations:** Department of Internal Medicine, Mayo Clinic, Jacksonville, FL, USA

## Abstract

Infiltrative cardiomyopathies include a variety of disorders that lead to myocardial thickening resulting in a constellation of clinical manifestations and eventually heart failure that could be the first clue to reach the diagnosis. Among the more described infiltrative diseases of the heart is amyloid cardiomyopathy. The disease usually presents with subtle, nonspecific symptoms. Herein, we illustrate a case of recurrent syncope as the initial presenting symptom for systemic amyloid with polyneuropathy and cardiomyopathy as a cause of syncope. The article illustrates the role of advanced cardiac imaging in the diagnosis of the disease with a focused literature review. We also highlight the role of early, shared decision-making between patient, family, and medical team in the management of cardiac amyloidosis.

## 1. Introduction

Infiltrative cardiomyopathies include a variety of disorders that lead to myocardial thickening, heart failure, and eventually death. Amyloid cardiomyopathy (ACM) represents the most commonly described infiltrative heart disease [1]. Amyloidosis comprises a group of heterogeneous systemic diseases characterized by formation of misfolded proteins that aggregate and deposit as *β*-pleated sheets [1]. Although many proteins can form amyloid deposits, the type of precipitated protein will define the subtype of amyloidosis, organs involved, and prognosis. Amyloid cardiomyopathy subtypes, organ involvement, and prognosis are illustrated in Table 1.

One of the first possible presenting symptoms of ACM is syncope with severe orthostatic hypotension. When syncope is encountered as the first symptom, it can be an ominous sign of advanced and severe disease of the myocardium [2, 3]. Therefore, early recognition of the disease is crucial to promptly initiate the appropriate treatment. Despite therapy, mortality rate in cardiac amyloidosis is high, and most patients with AL ACM suffer sudden death within one year from the initial diagnosis [4].

## 2. Case Presentation

A 63-year-old Caucasian woman presented to the emergency department with one year history of recurrent and progressive syncopal episodes rendering her bed-bound. The syncopal events were triggered by positional changes and were preceded by light-headedness. During the event, she had no gastrointestinal symptoms, urinary or bowel incontinence, tongue biting, or abnormal movements. After these episodes, she returned to baseline within a few seconds. She had an unintentional 10 kg weight loss over the course of her disease, and previous diagnostic workup including complete blood count, thyroid function tests, ACTH, electrocardiogram (EKG), echocardiogram, electroencephalogram and magnetic resonance imaging of the brain, computed tomography of the abdomen, upper endoscopy, and colonoscopy had been all unremarkable. Past medical history was notable for Hurthle cell carcinoma of the thyroid gland 4 years prior to the presentation for which she underwent thyroidectomy with no associated complications.

Her physical examination demonstrated cachexia, a supine blood pressure (BP) of 127/72 mmHg and a heart rate (HR) of 79 beats per minute (bpm), sitting BP of 93/60 mmHg and a HR of 91 bpm, and standing BP of 73/42 mmHg and a HR of 97 bpm. Her oral examination showed moist mucosa without macroglosia, lungs were clear and heart sounds were distant, there was no organomegaly and no peripheral edema, her skin had no bruises or periorbital purpura, and her neurological evaluation was unremarkable. Initial evaluation revealed normal cell count, hyponatremia of 132 mmol/L (135–145 mmol/L), creatinine of 0.6 mg/dL (0.8–1.3 mg/dL) with an eGFR of 129.9 ml/min/1.73 m^2^, serum albumin of 3.9 g/dL (3.5–5.0 g/dL), and NT-Pro BNP of 478 pg/mL (<185 pg/mL), and urinalysis was unremarkable. Protein electrophoresis revealed an M-spike in the gamma fraction (1.0 g/dL), urine immunofixation showed monoclonal lambda and IgG lambda fragments, serum-free light chains (FLC) were 7.29 mg/dL (0.5700–2.63 mg/dL), and a kappa/lambda ratio was 0.1550 (0.2600–1.65).

**Figure 1:**
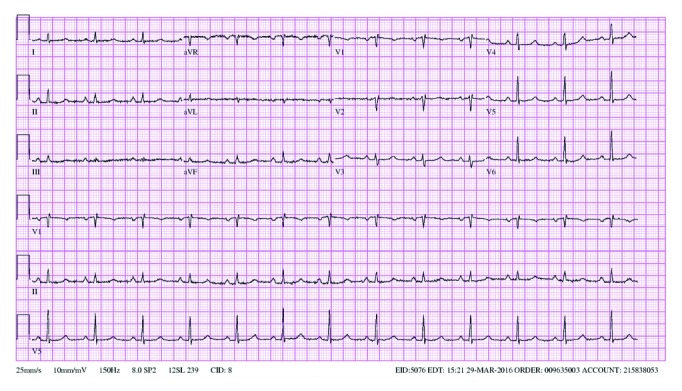
EKG demonstrating low-voltage criteria in most of the leads. Although the influence of the pericardial effusion is uncertain, this finding could be consistent with an infiltrative disease.

**Figure 2:**
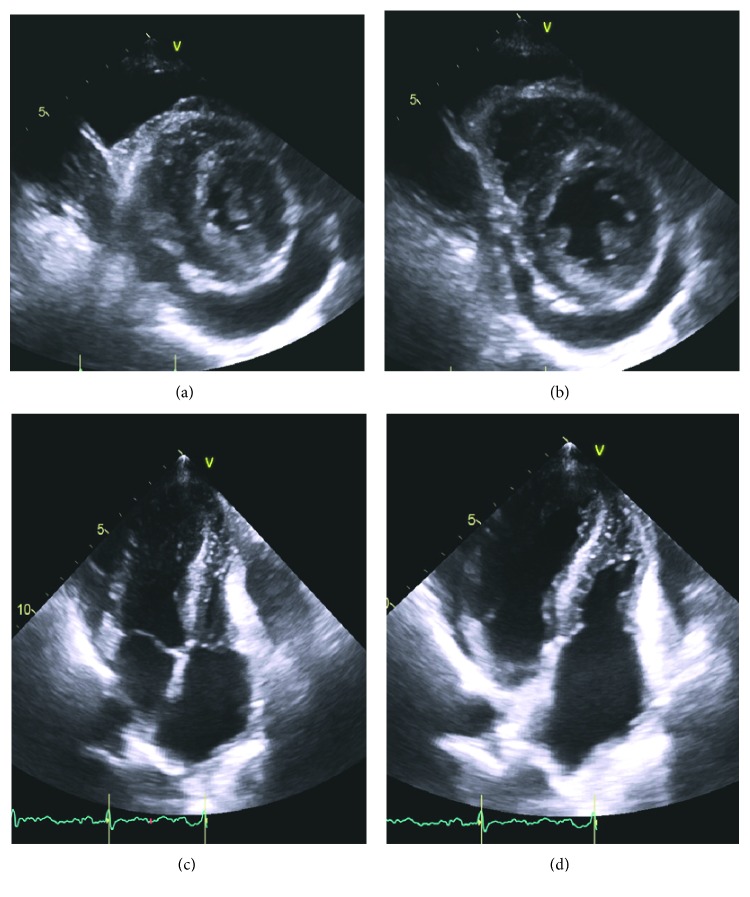
Transthoracic echocardiogram showing moderate-size pericardial effusion in both parasternal short axis view ((a) systole; (b) diastole) and parasternal long axis view ((c) systole; (d) diastole). Echocardiogram also demonstrates granular sparkling (speckled) appearance of myocardium, suggestive of cardiac amyloid.

**Figure 3:**
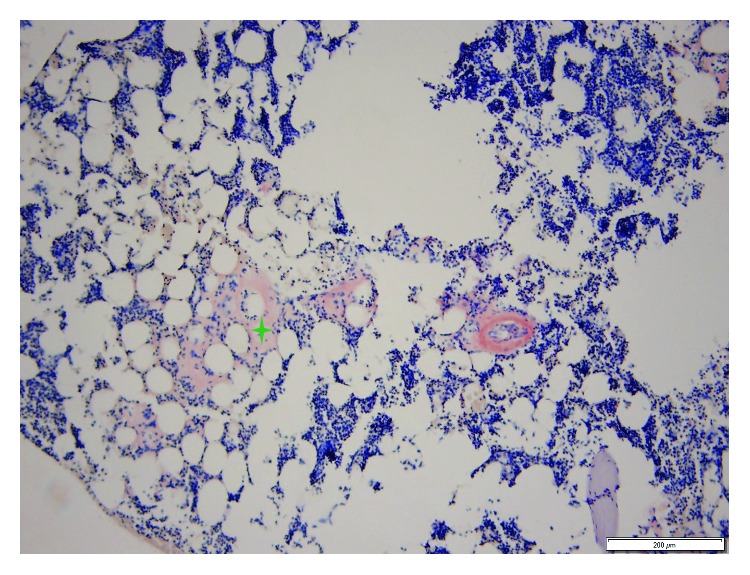
Bone marrow biopsy. Congo red 100x. Evidence of amyloid deposits.

**Figure 4:**
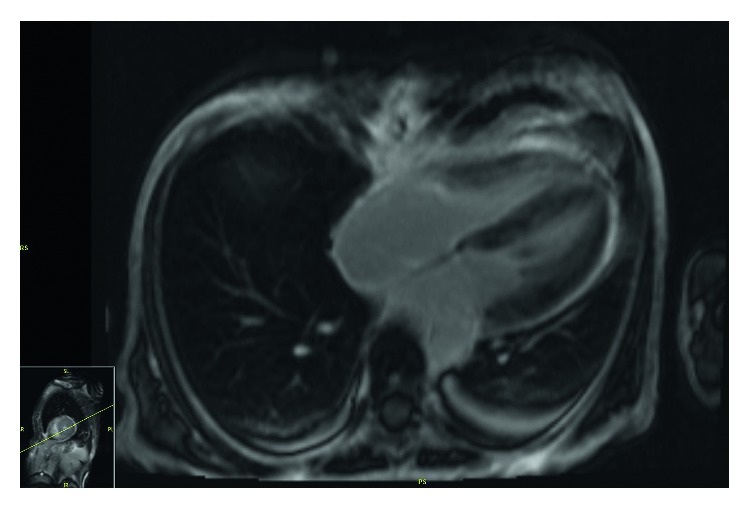
Cardiac MRI. Evidence of patchy focal late gadolinium enhancement of the pericardium with suboptimal nulling of the myocardium.

Her EKG showed sinus rhythm, signs of left atrial enlargement and low voltage in the majority of leads (Figure 1). A transthoracic echocardiogram demonstrated a large pericardial effusion, left ventricular ejection fraction of 73%, grade I diastolic dysfunction, left ventricular hypertrophy, abnormal longitudinal contraction, and a granular sparkling (speckled) appearance of the myocardium, findings suggestive of ACM (Figure 2). Bone marrow biopsy revealed 5% atypical plasma cells with lambda restriction, Congo red positive amyloid deposits, and mass spectrometry consistent with AL (lambda)-type amyloidosis (Figure 3). Cardiac magnetic resonance (CMR) demonstrated suboptimal myocardial nulling with patchy focal late gadolinium enhancement affecting the pericardium and atrial walls, a large pericardial effusion, and a pleural effusion suggestive of amyloid infiltration (Figure 4).

**Figure 5:**
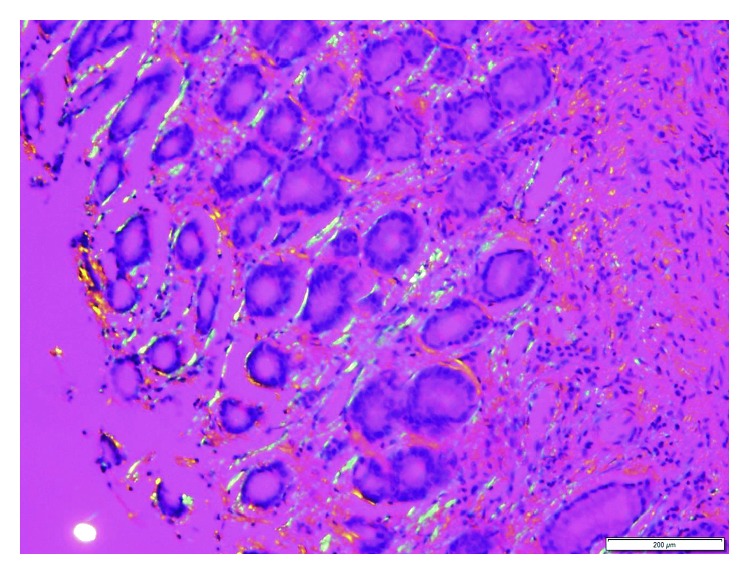
Pericardial biopsy. Congo red under polarized light 200x. Evidence of apple-green birefringence indicative of the presence of amyloid fibrils.

The size of the pericardial effusion was felt to be a contributor to the patient's positional syncopal episodes, and hence, a pericardial window and pericardial biopsy for diagnostic and therapeutic purposes was pursued. Results of this biopsy were consistent with deposition of amyloid, confirming the diagnosis of ACM (Figure 5). Mass spectrometry performed on this tissue yielded AL-type amyloid deposition.

A multidisciplinary team approach including internal medicine, hematology/oncology, cardiology, and palliative care medicine was adopted to treat the patient's condition. We aimed for shared decision-making with the patient and family regarding the management plan. The patient and her family elected initially to undergo an aggressive approach with chemotherapy. Cyclophosphamide, bortezomib, and dexamethasone (CyBorD) regimen was initiated.

The patient's BP failed to improve, posing an obstacle for appropriate medical management of heart failure. Treatment to support BP with midodrine was initiated along with treatment for systemic amyloidosis with CyBorD.

Despite the prompt initiation of treatment, her levels of lambda FLC progressively increased. Due to her poor response and intolerance to chemotherapy, it was decided to discontinue CyBorD and offer palliative care due to her overall poor prognosis. Two months after discontinuation of therapy and only eight months after the initial diagnosis was made, the patient died due to cardiogenic shock.

## 3. Discussion

Immunoglobulin AL-type amyloidosis is the most common form of amyloidosis affecting 10.5 persons per million in the United States [2]. It occurs as a consequence of abnormal proliferation of plasma cells within the bone marrow that overproduce and release lambda, and, less often, kappa-free light chains which deposit in different tissues including the myocardium [5]. Transthyretin (TTR), on the other hand, is a protein primarily synthesized by the liver, playing an important role in neurologic regeneration and cognition [6]. Its misfolding and secondary formation of amyloid deposits can occur as a consequence of autosomal dominant mutations (mutant ATTR (ATTR-mt) previously known as familial amyloidosis). Depending on the type of mutation, manifestations can range from cardiac involvement (familial cardiac amyloidosis), peripheral nerve damage (familial amyloid polyneuropathy), and overlapping phenotypes [4]. Importantly, TTR misfolding can also occur sporadically (wild-type (ATTR-wt) previously termed senile systemic amyloidosis). This type of amyloidosis, occurs almost exclusively (90%) in men older than 60 years old. Cardiac involvement in ATTR-wt presents as an isolated cardiomyopathy and carries a better prognosis than any other ACM with a median survival of 75 months [2, 4].

Immunoglobulin AL amyloid cardiomyopathy has an estimated annual incidence of 6 to 10 cases per million persons in the western countries [7]. The heart is the second most commonly involved organ after kidneys, affecting up to 50% of patients [2]. It occurs more often in men from the fifth to the seventh decade of life, and it is the leading cause of mortality in amyloid disease [5]. Usually multiple organs are affected at presentation; however, isolated cardiac disease has been observed in 4% of patients [5].

Amyloid cardiomyopathy has a wide spectrum of clinical presentations that can range from asymptomatic to sudden death. The most common initial symptom is exertional dyspnea followed by peripheral edema and ascites due to development of restrictive cardiomyopathy [2]. Angina (due to amyloid deposition in the coronary vasculature), palpitations, and atrial arrhythmias (due to conduction system abnormalities) can also occur [5, 8]. Although syncope has been identified in up to 14% of patients, in our literature review, only few cases have reported syncope as the presenting symptom in ACM [2, 7, 9]. Furthermore, its presence is often considered a marker of poor prognosis [2, 3]. The etiology for syncope can be multifactorial from arrhythmias, heart block due to conduction system disease [3, 7, 8], severe orthostatic hypotension, and autonomic neuropathy [2, 3, 5, 7, 8].

The diagnosis of ACM is usually a multistep process. Findings of EKG showing low voltage are a frequent but nonspecific marker of the disease, as it is also present in other infiltrative diseases [10]. However, the discrepancy between increased ventricular mass on echocardiogram and low voltage on EKG is highly suspicious for this pathology [2]. Other possible findings include tachycardia, pseudoinfarcts, or atrial fibrillation/flutter [7, 8]. Common echocardiographic findings include left ventricle hypertrophy, diastolic dysfunction, and abnormal long axis contraction [5]. The increased myocardial echogenicity as a consequence of amyloid infiltration has been described as speckling sign. Speckling is a granular sparkling appearance and has been reported to be present in up to 64% of the patients with ACM [8]. The systolic longitudinal strain is an echocardiographic technique that measures the longitudinal LV function. This method can help with the early diagnosis of amyloid cardiomyopathy and is a prognostic factor in patients with amyloidosis. It can be used to predict mortality and need for transplantation in this population, even in patients with preserved EF [11, 12]. Pericardial effusion is another common finding that is found in 43% of patients [8]. However, it is rarely associated with cardiac tamponade [2]. Finally, atrial dilation and valve thickening can also be present [5, 8]. Late gadolinium enhancement is useful for the diagnosis of ACM when seen on CMR [13, 14]. However, the presence of patchy focal late gadolinium enhancement or suboptimal myocardial nulling (lack of adequately nulled “black” images) can also be seen in early amyloidosis [14]. Importantly, while the absence of late gadolinium enhancement on CMR does not rule out cardiac amyloidosis, its presence is considered a marker of amyloid load which correlates with prognosis [2, 5, 13–16].

T1 mapping is a recent CMR technique that allows assessing the composition of the myocardium by quantifying the signal from the native tissue without the use of contrast (T1) [15, 16]. ACM causes an increased T1 which can be present even before wall thickening occurs [15]. This technique, though still under investigation, could potentially be used in those with renal impairment or as a complimentary image in those situations of ambiguous late gadolinium enhancement [16]. Technetium-derived tracers accumulate on myocardial amyloid deposits. Thus, myocardial scintigraphy can diagnose cardiac ATTR amyloidosis without histological confirmation as long as there is no plasma cell dyscrasia [15, 17]. Other amyloid-avid tracers like metaiodobenzylguanidine (MIBG) in conjunction with positron emission tomography (PET-CT scan) may assist with early detection of ACM and prognosis evaluation, as well as with objective evaluation of treatment responsiveness [18].

Immunofixation of urine and serum as well as immunoglobulin-free light chain have been traditionally used to identify the type of amyloid [19]. If AL amyloidosis is present, immunofixation usually demonstrates a monoclonal band, and serum-free light chains show an abnormal kappa/lambda ratio in more than 90% of patients [2]. Under these circumstances, a bone marrow biopsy should be done to rule out the presence of multiple myeloma [2]. However, a definitive AL amyloidosis diagnosis requires tissue biopsy. The use of endocardial or epicardial biopsies is not required in the presence of systemic amyloidosis as an extracardiac sample (e.g., abdominal fat pad) can be obtained [2, 5]. These should be prepared with Congo red stain to reveal the characteristic apple-green birefringence when viewed under polarized microscopy [2, 5]. Once amyloidosis is confirmed, defining the type of deposit should be pursued [19]. In addition to the initial immunofixation and free light chain testing which guides towards the type of amyloidosis, some authors suggest the use of mass spectroscopy on the tissue biopsy to clearly differentiate AL from TTR amyloidosis [19].

Another important step after diagnosing AL amyloidosis consists in identifying involvement of other organs. This is done by using a set of criteria proposed in 2005 and modified in 2010 [20, 21]. Cardiac involvement is defined by a pro-BNP > 332 ng/L or a mean wall thickness in diastole >12 mm in echocardiogram. Renal involvement is defined by the presence of >0.5 g of protein in a 24-hour urine sample. Liver involvement is defined by hepatomegaly or an alkaline phosphatase >1.5 times of upper normal value. Gastrointestinal involvement usually requires direct biopsy sampling to differentiate from autonomic dysmotility [21]. Pulmonary involvement can be determined by either a direct biopsy or by radiographic changes consistent with diffuse interstitial lung disease. However and as noted above, a direct organ biopsy is not required if another tissue biopsy has confirmed the diagnosis [21]. To determine if nerves or soft tissue is involved, clinical symptoms should be used as no testing is helpful.

Besides the presence of late gadolinium enhancement, high levels of N-terminal natriuretic peptide B, troponins, and free light chains, as well as the presence of pleural effusion, have all been found to be negative prognostic markers in AL amyloidosis [2, 5, 12]. Treatment of AL cardiac amyloidosis is directed to manage heart failure symptoms and treat the underlying amyloidosis. For the former, the cornerstone of management is the use of low-dose diuretics. Other heart failure medications including angiotensin-converting enzyme inhibitors, angiotensin-receptor blockers, beta-blockers, and calcium-channel blockers may worsen the hypotension and are poorly tolerated [2, 5, 7]. For orthostatic hypotension, graduated compression stockings and midodrine are commonly used [5]. Management of the underlying cell dyscrasia requires the use of chemotherapeutic agents. Specifically bortezomib in combination with dexamethasone and cyclophosphamide (CyBorD) has considerably improved the survival of these patients [22]. Autologous stem-cell transplantation (ASCT) can be attempted in suitable patients in whom other chemotherapeutic agents have failed. Of note, although increasing age is often associated with multiple comorbidities, it is not considered a contraindication to therapy, and rather other parameters, such as performance status, are considered in this decision [23, 24]. Treatment of TTR-mt cardiac amyloidosis includes the use of liver transplantation in an attempt to clear the defective TTR. As the TTR is native in patients with TTR-wt, liver transplantation is not used in this population [5]. TTR stabilizers like tafamidis (approved in the European Union and Japan for ATTR-mt) and diflunisal are being investigated on multiple clinical trials [5]. The use of agents able to silence the gene expression of TTR by the liver as a way to block hepatic synthesis is also under investigation [5]. Finally, doxycycline has also proven to promote fibril disaggregation and is also part of different clinical trials [5].

Since amyloid deposition can occur in the newly acquired organ, heart transplantation in AL amyloidosis may be of limited utility if the underlying plasma disorder is not addressed. Thus, the use of adjuvant chemotherapy can improve the 5-year survival from 20% to 37% [25, 26]. More recently, the use of heart transplantation in conjunction with ASCT in selected patients has increased the 5-year survival to 65% [25]. The use of heart transplantation with liver transplantation has been associated with good outcomes in patients with ATTR-mt [25]. On the other hand, only few cases of heart transplantation have been used on ATTR-wt given the advanced age at presentation, however, has shown some success [25].

Despite advancements in treatment and diagnosis, the prognosis of AL amyloid cardiomyopathy remains overall poor. As opposed to ATTR-wt where survival averages 75 months, it is estimated that only 29% of patients survive one year and the median survival decreases to 6 months if heart failure is left untreated [2, 5, 27]. Sudden cardiac death occurs in the majority of these patients secondary to pulseless electrical activity, bradyarrhythmias, and asystole and therefore the indications of implanted cardiac defibrillator in these patients remain unclear [27, 28]. For this reason, palliative care should be involved early in the plan of care, and many may meet criteria for hospice early in the course of the disease. Although few studies are available, palliative care and symptomatic management for these patients have recently been reviewed and recommended [29].

Based on our experience in the treatment of this patient, we recommend the early involvement of patients and their families in the management plan by shared decision-making and providing a realistic view on the disease prognosis. Also, we recommend the early involvement of palliative care medicine service as part of the multidisciplinary approach to treat patients with this complex disease.

## 4. Conclusion

Cardiac amyloidosis is a complex disease that can rarely present as syncope. Its diagnosis can be challenging as even specialized testing such as delayed gadolinium enhancement in cardiac magnetic resonance imaging can lack sensitivity. Few nuclear imaging tracers under investigation may provide a promising role in the diagnosis of amyloid cardiomyopathy. When suspected, a bone marrow biopsy should be done to rule out the presence of multiple myeloma; however, the only way to confirm the diagnosis is with tissue biopsy. Management is directed at control heart failure symptoms and the underlying process causing amyloid deposition. As a consequence, prognosis is overall poor and the majority of patients with AL amyloidosis suffer sudden cardiac death within one year. Therefore, we recommend the early involvement of palliative care as part of the multidisciplinary approach in managing patients with amyloid cardiomyopathy.

## Figures and Tables

**Table 1:** 

Type of amyloidosis	Amyloid composition	Organs involved	Symptoms	Prognosis	Other
AL amyloidosis	Immunoglobulin-derived light chain	Kidneys > heart, gastrointestinal tract, nervous system	Left and right heart failure, syncope, and autonomic neuropathy	Median survival 8–12 months	Plasma cell dyscrasia, requires treatment
ATTR-mt (familial)	Mutant transthyretin	Heart and nervous system	Heart failure can be severe, ±neuropathy	Varies depending on mutation, but favorable compared to AL	Autosomal dominant
ATTR-wt	Wild-type transthyretin	Heart	Less severe heart failure than AL or ATTRm	75 months	90% are men > 60 years old

AL = amyloid light chain; ATTR-mt = mutant transthyretin amyloidosis; ATTR-wt = wild-type transthyretin amyloidosis.
